# GPCR-SAS: A web application for statistical analyses on G protein-coupled receptors sequences

**DOI:** 10.1371/journal.pone.0199843

**Published:** 2018-07-25

**Authors:** José Carlos Gómez Tamayo, Mireia Olivella, Santiago Ríos, Marlous Hoogstraat, Angel Gonzalez, Eduardo Mayol, Xavier Deupi, Mercedes Campillo, Arnau Cordomí

**Affiliations:** 1 Laboratori de Medicina Computacional, Unitat de Bioestadística, Facultat de Medicina, Universitat Autònoma de Barcelona, Barcelona, Spain; 2 Grup de recerca de Bioinformàtica i Estadística Mèdica, Universitat de Vic, Barcelona, Spain; 3 Department of Molecular Carcinogenesis, The Netherlands Cancer Institute, Amsterdam, The Netherlands; 4 Laboratory of Biomolecular Research and Condensed Matter Theory Group, Paul Scherrer Institute, Villigen, Switzerland; Cincinnati Children's Hospital Medical Center, UNITED STATES

## Abstract

G protein-coupled receptors (GPCRs) are one of the largest protein families in mammals. They mediate signal transduction across cell membranes and are important targets for the pharmaceutical industry. The G Protein-Coupled Receptors—Sequence Analysis and Statistics (GPCR-SAS) web application provides a set of tools to perform comparative analysis of sequence positions between receptors, based on a curated structural-informed multiple sequence alignment. The analysis tools include: (i) percentage of occurrence of an amino acid or motif and entropy at a position or range of positions, (ii) covariance of two positions, (iii) correlation between two amino acids in two positions (or two sequence motifs in two ranges of positions), and (iv) snake-plot representation for a specific receptor or for the consensus sequence of a group of selected receptors. The analysis of conservation of residues and motifs across transmembrane (TM) segments may guide the design of more selective ligands or help to rationalize activation mechanisms, among others. As an example, here we analyze the amino acids of the “transmission switch”, that initiates receptor activation following ligand binding. The tool is freely accessible at http://lmc.uab.cat/gpcrsas/.

## Introduction

G protein-coupled receptors (GPCRs) are one of the most prevailing protein families in mammalian genomes [1] and the major protein family as drug targets, with about one third of marketed compounds targeting them [2, 3]. They are involved in most signal transduction processes across membranes, including the response to hormones and neurotransmitters and the senses of sight, smell and taste. GPCRs transduce extracellular signals across the cell membrane through G protein dependent but also through G protein independent processes [4]. GPCRs are classified into six families or classes named A to F according to sequence similarities [5–7]. Only classes A, B, C and F are present in humans and class A or rhodopsin-like comprises, by far, the largest number of members [8]. The available GPCRs crystal structures showed a conserved TM structure with a common fold formed by an extracellular N–terminus, seven transmembrane helices (TM1-7), connected by alternating intracellular (ICL1 to ICL3) and extracellular (ECL1 to ECL3) hydrophilic loops, and a cytoplasmic C–terminus [9–11] that starts with an α-helix (Hx8) parallel to the cell membrane in classes A, B and F. This structural similarity in the TM domain facilitates comparative analysis between members of the family. This was early recognized by Ballesteros and Weinstein when they developed the common residue numbering system [12], and has been exploited in the GPCRdb [13], a dedicated database for GPCRs, with sequence, structural and ligand information.

Here we present GPCR-SAS, a web application that permits easy comparison and statistical analysis of sequence positions or motifs within the TM helices and helix 8 across GPCRs of classes A, B, C and F. Our tool can be of help in identifying residues undergoing correlated evolution, and thus represents a useful instrument to rationalize ligand selectivity, G protein recognition or receptor activation, among others. As an example, here we illustrate the utility of GPCR-SAS by analyzing the amino acids that belong to the “transmission switch”, that initiates receptor activation following ligand binding [14–16].

## Material and methods

### GPCR-SAS web application

GPCR-SAS is a web application freely accessible at http://lmc.uab.cat/gpcrsas/. The main tool is written in Python (version 2.7; available at http://www.python.org) and employs Django framework (version 1.5; available at https://djangoproject.com). The application relies on a MySQL database (version 5.1.73; available at https://www.mysql.com/) that contains the sequence alignments of all GPCRs sequences and previously reported classification schemes [8, 13, 17–19]. Its design and implementation permit automatic incorporation of additional sequences as they are incorporated or edited in the UniProt [20].

**Fig 1:**
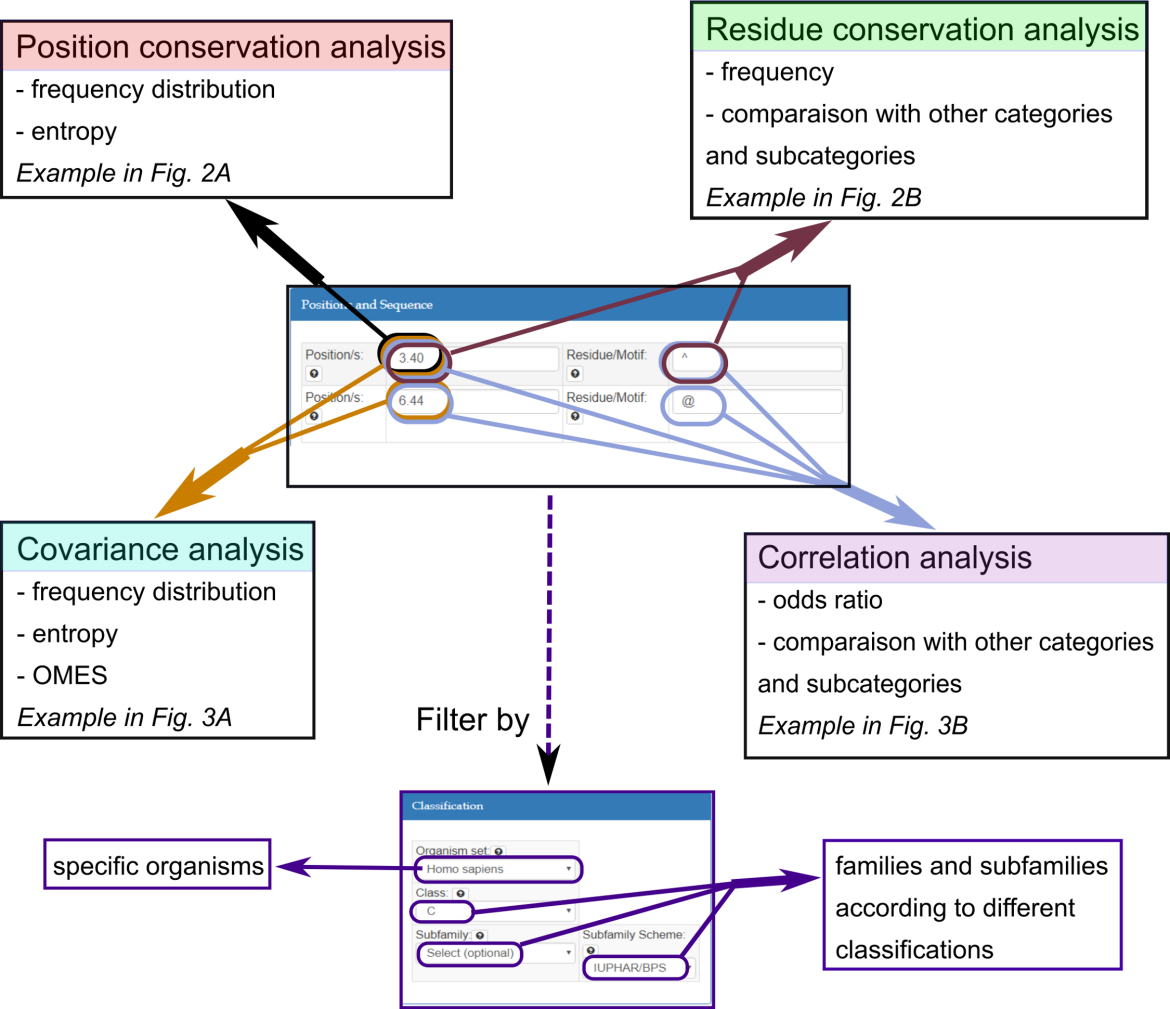
Schematic representation of the input forms of GPCR-SAS and the possible outcomes. The input of GPCR-SAS consists in two main sections: (i) Positions and Sequence: where the user can introduce a position/set of positions and a residue or sequence motif and (ii) Classification, that provides navigable multilevel hierarchical classification of GPCRs in families, branches and various levels of subfamilies according to different implemented schemes.

The input of GPCR-SAS consists in two main sections: (i) *Positions and Sequence*: where the user can introduce a position/set of positions and a residue or sequence motif and (ii) *Classification*, that provides navigable multilevel hierarchical classification of GPCRs in families, branches and various levels of subfamilies according to different implemented schemes. The output provides conservation, covariance or correlation analysis for different classification sub-levels depending on the input provided, as schematically shown in Fig 1.

### Database of aligned GPCR sequences

GPCRS-SAS database currently contains multiple sequence alignments of the TM helices and helix 8 of 2982 GPCRs sequences of all species: 2377 class A, 206 class B, 111 class C and 297 class F. Amino acid sequences for all GPCRs belonging to classes A, B, C and F were retrieved from the UniProtKB/Swiss-Prot database (http://www.uniprot.org) on May the 15^th^ 2018 [20]. For each class, the sequences of receptors with an available crystal structure were aligned in a first step using MultiProt [21]. This structural alignment was used to define consensus boundaries for each TM helix on every GPCR class and to construct an initial sequence profile aware of the TM segments. Because the main purpose of GPCR-SAS is the comparison between sequences, we chose to be conservative in terms of gap introduction. Otherwise the alignment cannot be used for unambiguous predictions [22]. In the class A, irregularities observed between different structure patterns were handled using gaps on TMs 2 and 5 as previously described [23–25]. For each class, we aligned the sequences in three steps: first human sequences, next the rest of mammalian sequences and finally, the remaining vertebrate and invertebrate sequences. All multiple sequence alignments were performed with Clustal Omega [26]. At each step the alignment was manually curated ensuring lack of gaps on TM regions other than those at TMs 2 and 5 in class A receptors [23, 24] or those associated to trivial deletions. Finally, the alignments for each class were assembled based on the structural alignment between crystallized receptors from the different classes [27, 28]. The final alignment is similar to the one presented by Cvicek and collaborators [22] except for the gaps in TMs 2 and 5 (positions 2x551 and 5x461 according to the generic residue numbering [25]), whereas it does not contain other gaps that appear in the GPCRdb alignment [25].

The updated list of GPCRs sequences and the alignment are available at the help panel (http://lmc.uab.cat/gpcrsas/about/). By default, the class A set in the GPCRS-SAS database consists of the 1824 non-olfactory GPCRs, since olfactory receptors are excluded to avoid biasing of results towards this subfamily—it accounts for almost two thirds (561 receptors) of the human class A GPCRs. The sequence alignment for TM helices and the short helix 8 perpendicular to de membrane for a selected group of receptors is shown in S1 Fig. Regular updates are planned every 3 months. This step implies aligning new sequences to the previous alignment and assigning the different classification categories. We have scheduled a yearly full update where we will rebuild the complete alignment to account for possible changes in the structural alignment due to new structures.

### Sequence numbering scheme

Each position in the TM segments is numbered according to the Ballesteros & Weinstein numbering scheme for class A GPCRs [12]. In this numbering, the position of each residue is described by two numbers: the helix in which the residue is located and the position relative to a conserved residue in that helix, arbitrarily assigned to 50, separated by a dot. In class A, these amino acids are: N1.50 in TM1 (97.6% conserved in human class A excluding olfactory receptors; data from GPCR-SAS), D2.50 in TM2 (92.1%), R3.50 in TM3 (94.8%), W4.50 in TM4 (95.8%), P5.50 in TM5 (76.0%), P6.50 in TM6 (98.3%), and P7.50 in TM7 (93.7%). Although GPCRs of classes other than A most often do not have such conserved amino acids at these positions [25], extrapolation of the class A numbering-scheme is now possible thanks to structure-based sequence alignment between classes [27, 28]. S1 Fig shows the sequence alignment for the TM helices of a selected group of receptors from different classes.

### GPCR classifications

GPCR-SAS queries permit to filter receptor sequences based on previously reported classification systems including: (i) Fredriksson [8], based on a phylogenetic analysis of human GPCR sequences (branches and one subfamily level); (ii) GPCRdb [29], which uses a pharmacologic classification of the receptors (three subfamily levels); (iii) BIAS-PROF GDS [17], based on the comparison of the protein sequence using the physicochemical properties of the amino acids (two subfamily levels); (iv) Rognan [18], that relies on the phylogenetic analysis of 30 positions putative involved on the ligand binding site (one subfamily level), (v) Chabbert [19], which employs multidimensional scaling to cluster GPCRs (branches and one subfamily level), and (vi) GPCR SARfari (https://www.ebi.ac.uk/chembl/sarfari/gpcrsarfari), based on chemogenomic data (three subfamily levels). As a complementary filter option, we defined five sequence sets based on different levels of taxonomic classification: Human, Mammals, Vertebrates, Eukaryotes and All (sequences for all species).

## Analysis tools

The relevance of GPCR-SAS is its capability to analyze conservation of residues or sequence motifs across TM segments of GPCRs and to identify covariance between two positions or correlation between specific amino acids for various classification schemes at different classification levels (class, branch and subfamilies) and within five different taxonomic sets.

### Conservation analysis

The entropy can be given for a single position (i.e. 3.50) or for a range of consecutive (i.e. 3.50–3.54) or non-consecutive (3.50, 4.50, 5.50) positions. When the input is a single position, a graph with the amino acids counts is also displayed. For each position or range of positions *i*, the entropy of the information contained *H(i)* is computed according to Shannon’s theorem [30] as:

$$H(i)=-\sum_{x}^{}p_{x}(i)\log_{b}p_{x}(i)$$ (1)

where *p*_*x*_*(*_*i*_*)* is the probability mass function for the amino acid(s) at position (or group of positions) *i*. The logarithm base *b* serves to scale the entropy in the range [0, 1] for one or more positions. Consequently, *b* is *20*^*n*^, with *n* being the number of positions used for the calculation. A position or group of positions with low variability (high conservation) has an entropy *H(i)* close to 0, while high variability (low conservation) gives an entropy close to 1.

The percentage of occurrence of an amino acid or motif in a specific position(s) (i.e. N 7.49, P 7.50) can also be computed. In this case, the output allows comparison to other categories and subcategories. To represent residues or motifs with specific physicochemical properties the user can utilize one-character wildcards as a residue or as part of a motif with the following correspondences: ‘X’ (any amino acid), ‘@’ (aromatic, W/Y/F/H), ‘~’ (apolar, I/L/V/A/F/P), ‘+’ (positively charged, R/H/K), ‘-’ (negatively charged, D/E), ‘*’ (charged, R/H/K/D/E) and ‘^’ (polar, D/E/N/Q/K/R/H/S/T/C/W/Y).

### Covariance analysis

To analyze the covariance of two positions, GPCR-SAS uses the Observed Minus Expected Squared (OMES) [31] that is based on a χ^2^ test, and a corrected mutual information method (MIp) [32]. Both methods have previously been employed by Pelé et al. to identify evolutionary hubs between pairs of residues in GPCRs [33].

OMES calculates the difference between the observed and expected frequencies of each possible pair of amino acids or groups of amino acids *(x*, *y)*, at positions *i* and *j* of the alignment:

$$OMES(i,j)=\frac{1}{N(i,j)}\sum_{x,y}{\left(N_{x,y}^{obs}(i,j)-N_{x,y}^{\exp}(i,j)\right)}^{2}$$ (2)

with *N*(*i*,*j*), $N_{x,y}^{obs}$ and $N_{x,y}^{\exp}$ being the number of sequences in the alignment with non-gapped residues, the observed frequency and the expected frequency, respectively, at positions (or list of positions) *i* and *j*.

The *MI* content *MI(i*,*j)* between two positions (or lists of positions) *i* and *j* on an alignment is based on the probability of joint occurrence of events and is defined as:

$$MI(i,j)=\sum_{x,y}p_{x,y}(i,j)\ln\frac{p_{x,y}(i,j)}{p_{x}(i)p_{y}(j)}$$ (3)

where *p*_*x*_*(i)*, *p*_*y*_*(j)* and *p*_*x*,*y*_*(i*,*j)* are respectively the frequencies of amino acid *x* at position *i*, amino acid *y* at position *j* and the amino acid pair *(x*, *y)* at positions *i* and *j*.

The corrected MIp version is defined as:

$$MIp(i,j)=MI(i,j)-\frac{\frac{1}{n-1}\sum_{j\neq i}MI(i,j)\frac{1}{n-1}\sum_{i\neq j}MI(i,j)}{\frac{2}{n(n-1)}\sum_{i,j}MI(i,j)}$$ (4)

with *n* being the number of columns in the alignment.

To evaluate the statistical significance for the computed OMES and MIp values, GPCR-SAS provides the Z-scores and the associated p-values, which are computed by comparing with the mean value for all combinations of two positions.

### Correlation analysis

To determine the correlation between two sequence positions, the occurrence of the amino acid or motif at the first position or range of positions is associated with the occurrence of the amino acid or motif at the second position or range of positions. The occurrences are used to compute an odds ratio (and the associated 95% confidence interval) that estimates how strongly the presence/absence of one of the first amino acids or motif is correlated with the presence/absence of the second amino acid or motif. To facilitate the comparison with the other categories at the chosen level of classification and in the subcategories, the output of a correlation analysis also returns the same analysis for these groups.

### Snake-plot representations

GPCR-SAS can also provide snake-plot representations for the sequence of a specific receptor or for the consensus sequence of a group of receptors (S2 Fig). Each residue is represented by a circle with a letter in gray-gradient (representing the frequency of the residue on the class) and an outline in blue-gradient (representing the frequency of the residue on the selected group of receptors). In single-receptor snake-plots, residues colored in green are those that do not match the most conserved residue for the selected group of receptors (i.e. the selected subfamily).

## Results

**Fig 2:**
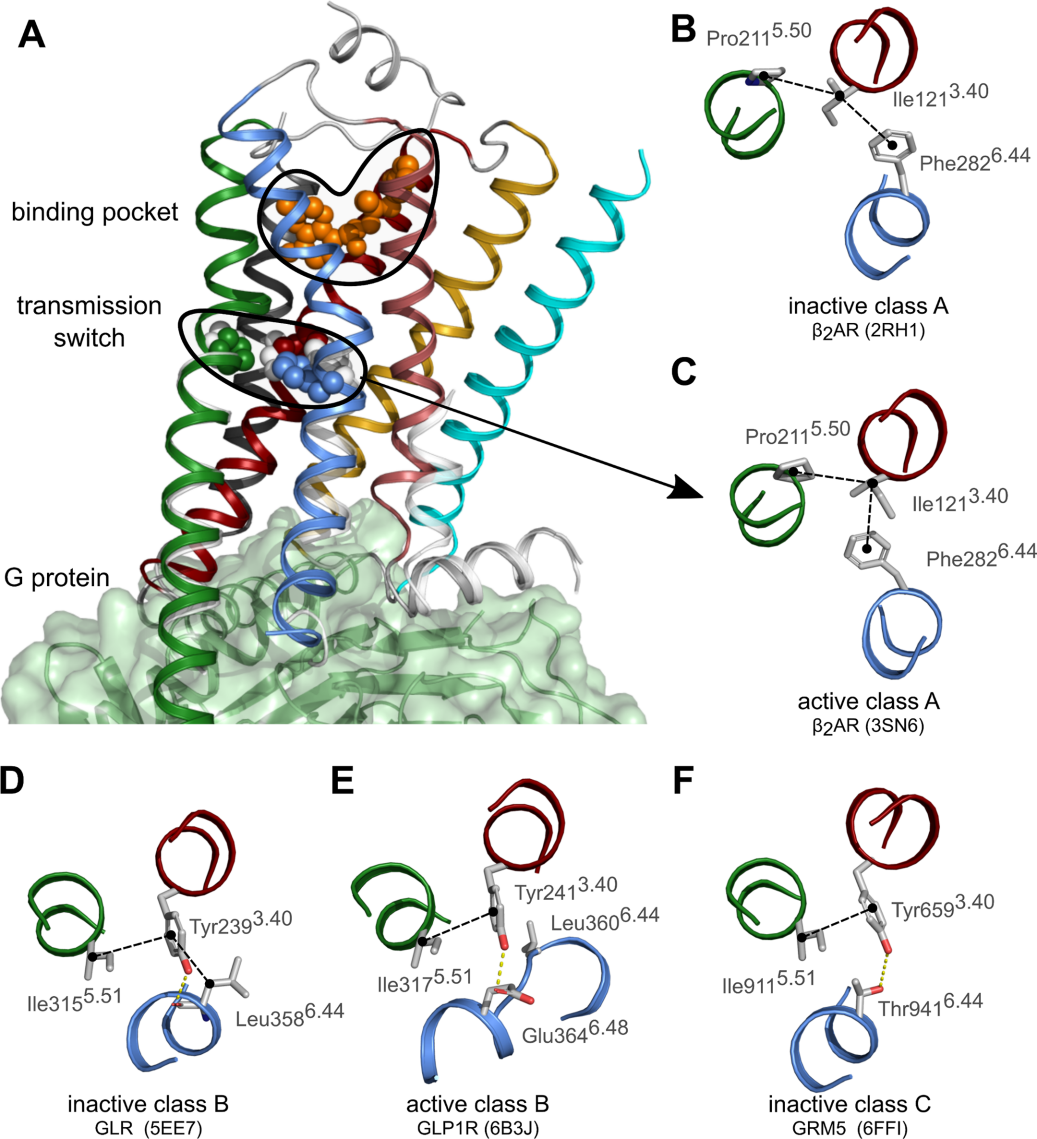
The transmission switch in the crystal structures of GPCRs. **(A)** Cartoon representation of the crystal structure of the active β_2_-adrenergic receptor (PDB id 3SN6) in complex with the G protein (green surface) illustrating the localization of the transmission switch just below the orthosteric binding pocket. The sidechains of residues of the transmission switch (3.40, 5.50 and 6.44) and the ligand (in orange) are shown as spheres. The color-code for the TM helices is 1:cyan, 2:gold, 3:red; 4:dark-gray, 5:green, 6_blue, 7:pale-red. Helix 8 and loops are shown in light-gray. Superposed (in white) the cytoplasmic ends of TMs 5, 6 and 7 and the residues of the transmission switch in the inactive structure (PDB id 2RH1). **(B-F)** Detail of the “transmission switch” in the crystal structures of inactive **(B)** and active **(C)** β_2_-adrenergic receptor (ADRB2), inactive glucagon receptor (GLR) **(D)**, active glucagon-like peptide 1 receptor (GLP1R) **(E)**, and inactive metabotropic glutamate receptor (GRM5) **(F)**. In receptors of classes B and C, residue 3.40 does not contact the residue at position 5.50 but rather the residue at position 5.51. **(E-F)** Dashed black lines indicate hydrophobic contacts whereas dashed yellow lines indicate hydrogen bonds.

To illustrate the use of GPCR-SAS, we analyzed conservation, covariance and correlation of the residues of the “transmission switch”, which is one of the initial steps of receptor activation following ligand binding in class A GPCRs [14–16]. The “transmission switch” involves amino acids at positions 3.40, 5.50 and 6.44. Rearrangement of the packing between these residues following ligand binding at the extracellular side of the receptor weakens the interface between TM helices 5 and 6 and triggers local conformational changes that are transmitted towards the cytoplasmic side, where G proteins and β-arrestins bind [34, 35] (Fig 2A). By using GPCR-SAS we will determine if the “transmission switch” is likely to exist in receptors belonging to classes B and C of GPCRs.

### The transmission switch in Class A GPCRs

**Fig 3:**
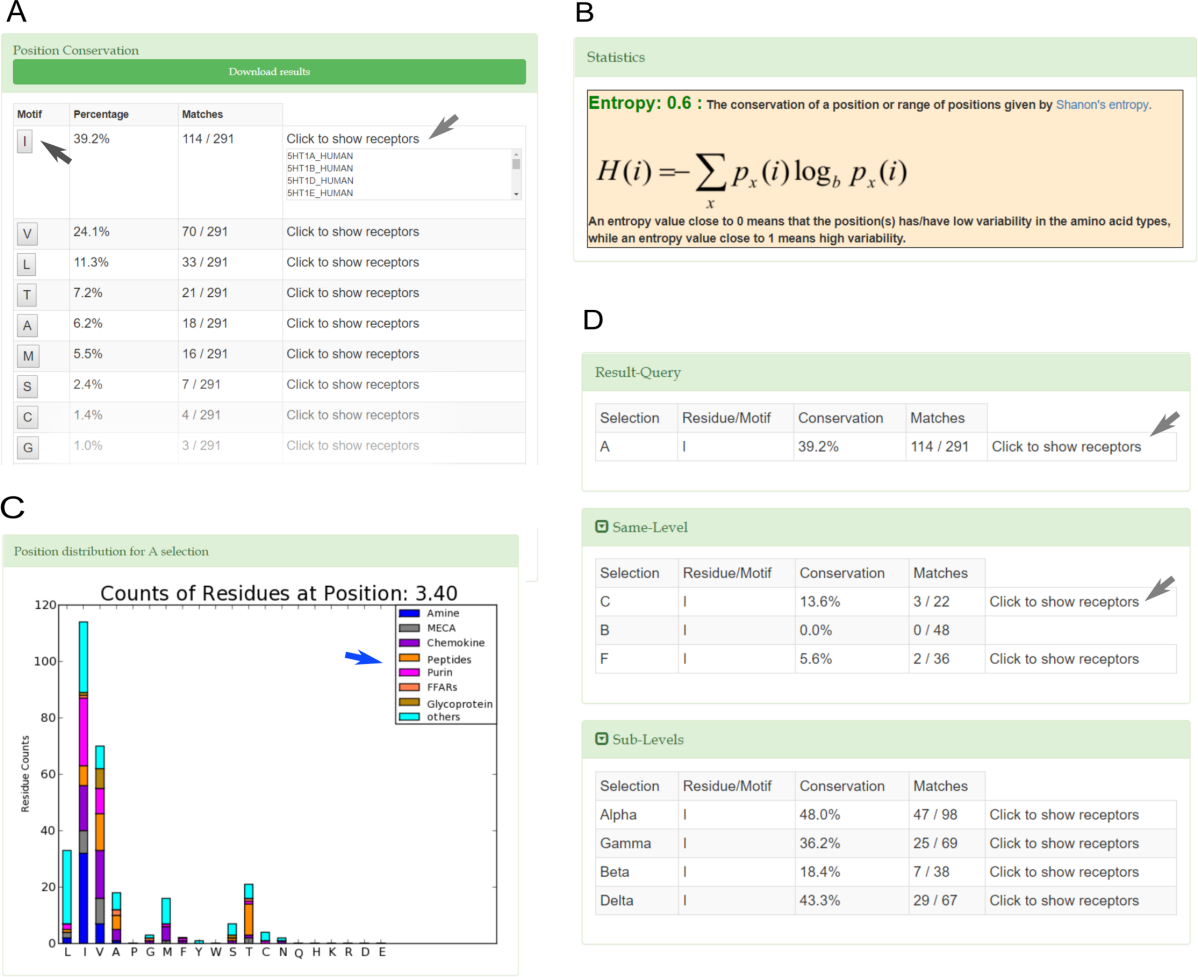
**Conservation analysis queries for position 3.40 (A-C) and for Ile at position 3.40 (D) in human non-olfactory class A GPCRs**. **(A)** the frequencies of amino acids at position 3.40; **(B**) the histogram of the amino acid frequencies at position 3.40 for the most the major subcategories (blue arrow); **(C)** the entropy of position 3.40; (**D**) the frequencies of Ile at position 3.40 for class A GPCRs compared the other classes (B, C and F; Same Level panel) and for the different class A branches (Sub-Level panel). Gray arrows indicate that it is possible to get the list of receptors that contain a certain residue or motif; the black arrow indicates that a click on the residue type in (**A)** provides the output displayed in (**D)**.

In class A GPCRs, the comparison between crystal structures of inactive versus active states showed a rearrangement of residues 3.40, 5.50 and 6.44 of the “transmission switch” [34, 36]. The changes observed in the β_2_-adrenergic receptor (β_2_-AR) are displayed in Fig 2B and 2C. The percentage of occurrence of each amino acid at position 3.40 for human non-olfactory class A GPCRs computed with GPCR-SAS is shown in Fig 3A. The information displayed includes also the entropy of the position (as a measure of variability, see Methods) and a histogram with the counts of each amino acid at this position grouped according to subfamily categories. The most prevalent residue in this position is IIe (39.2% conserved), followed by Val (24.1%) and Leu (11.3%), all of them sharing hydrophobic properties. The entropy value of 0.6 indicates a moderate variability (entropy values ranges between 0 and 1, see Methods). The histogram shows that most amine receptors, including the β_2_-AR, feature Ile at this position. Indeed, the frequency of Ile raises to 76.2% when restricting the query to the amine subfamily (according to Fredriksson’s classification scheme [8]). The output also provides (“Click to show receptors” button) the list of UniProt entry names for the receptors that matched the query. Clicking on an amino acid allows easy comparison with the same position in the other categories at the same level of classification (Same-level button) and within the categories of the child subfamilies (Sub-level button). In this particular example, the categories at the same level mean the other receptor classes (B, C and F) and the child categories are Fredriksson’s branches [8]. The output of the query for Ile (shown in Fig 3D) indicates that this residue is rarely/never found at position 3.40 in classes B (0%), C (13.6%) and F (5.6%). Regarding the sublevels, GPCR-SAS tells that Ile is preferentially found at the α (48.0%) and δ branches (43.3%). To expand the initial search, we used the wildcard for apolar amino acids (see Methods) which also accounts for Leu, Val, Ala, Phe and Pro. The frequencies for apolar residues add to 86.9% in class A, 79.2% in class B, 50% in class C, 69.4% in class F GPCRs. Thus, this analysis shows that despite Ile3.40 is not conserved in GPCRs of classes B, C and F, these receptors have mostly kept hydrophobic amino acids. Similar queries for the content of positions 5.50 and 6.44 in class A GPCRs reveals that Pro (78.4%) and aromatic residues (Phe:80.4%, Tyr:6.9%), respectively, are the most prevalent amino acids. The β_2_-AR has both Pro5.50 and Phe6.44 (see Fig 2B and 2C). To identify the most common residue triad, the three positions for human non-olfactory class A GPCRs were used as query (coma-separated in the box position). GPCR-SAS returns that the most frequent “transmission switch” residues are Ile3.40-Pro5.50-Phe6.44 as in the β_2_-AR (32.3%), Val3.40-Pro5.50-Phe6.44 (16.5%), and other triads with a percentage of occurrence smaller than 5% each. Overall, the triad Ile/Val3.40-Pro5.50-Phe6.44 comprises nearly half of the class A GPCRs.

### Transmission switch in classes B and C GPCRs

Next, we analyzed positions 3.40, 5.50 and 6.44 in GPCRs of classes B and C. GPCR-SAS shows that human class B GPCRs mostly feature aromatic residues at position 3.40 (Tyr:66.7% and Phe:20.8%), aliphatic residues at position 5.50 (39.6% Ile, 20.8% Val, 14.6% Ala) and Leu (64.6%) or Phe (20.8%) at position 6.44. Compared to class A GPCRs, class B receptors have switched residue types at positions 3.40 and 6.44 (from aliphatic-aromatic in class A to aromatic-aliphatic in class B), but still exhibit conserved residue types in these positions in this class. The results are compatible with our recent proposal, based on mutagenesis studies, that residues Phe3.40 and Leu6.44 form the “transmission switch” in the corticotropin-releasing factor 1 receptor [28]. The crystal structures of class B GPCRs show that the side chain of Tyr/Phe3.40 interacts with Leu/Phe6.44 in the inactive state but that this interaction is lost in the active state (Fig 2D and 2E). In addition, in the glucagon family of receptors (which contain Tyr3.40) the hydroxyl group of Tyr forms a hydrogen bond with the backbone of residue 6.44 in the inactive state and with the side-chain of Glu6.48 in the active state. GPCR-SAS tells that Glu6.48 is exclusive of the glucagon family of receptors. The most common pair for positions 3.40–6.44 is Phe/Leu. Class B receptors also lack the characteristic Pro at position 5.50 present in class A GPCRs.

**Fig 4:**
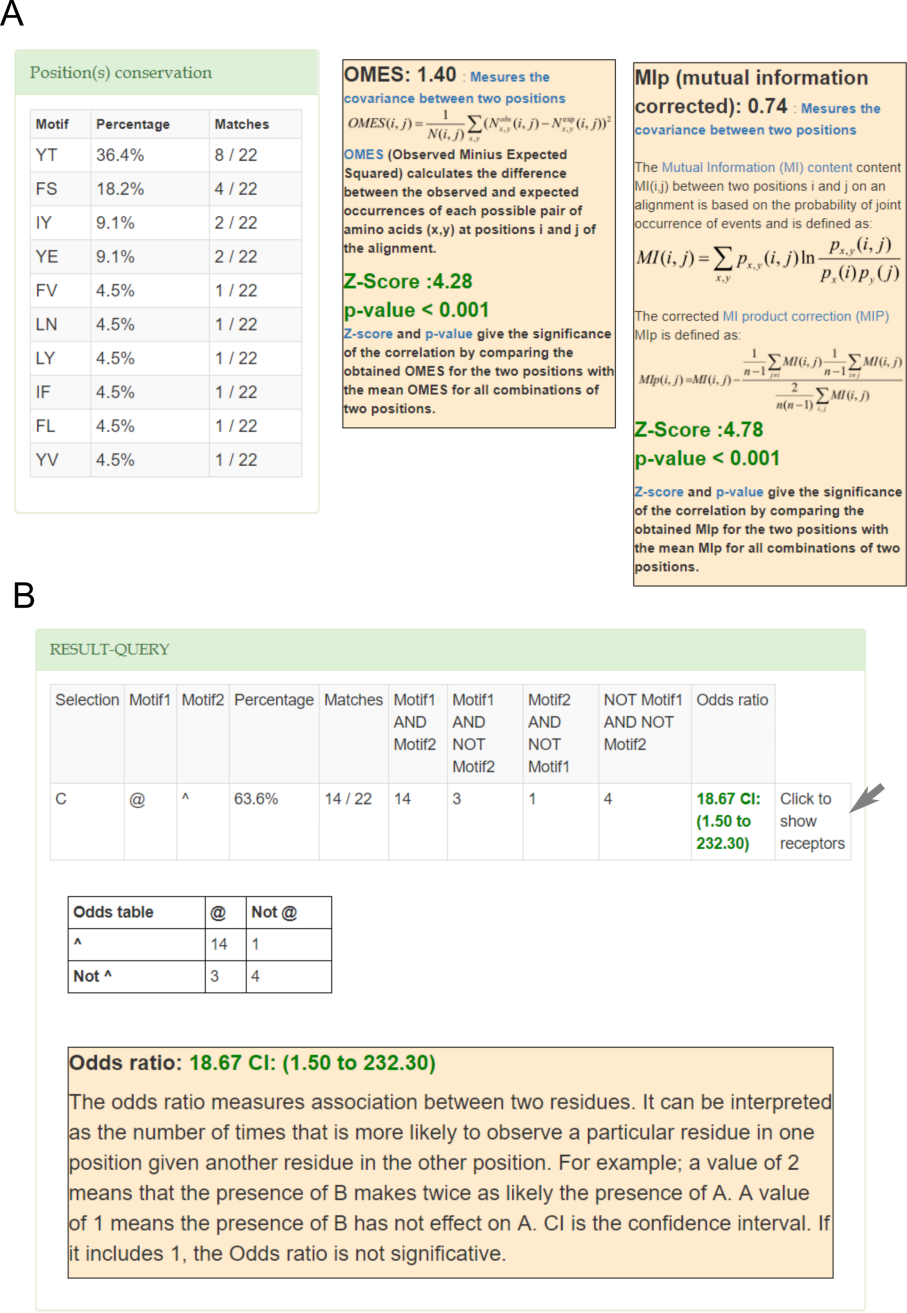
**Covariance (A) and correlation (B) analysis for positions 3.40 and 6.44 in class C GPCRs (A).** In (**A**) the left panel shows a list of all motifs and its frequencies and the right panel shows the statistical tests: the OMES and MIp analyses indicates statistical significant covariance between both positions. In (**B**) the odds ratio for an aromatic residue at position 3.40 and a polar residue at position 6.44, which indicates statistical significant correlation. The gray arrow indicates that it is possible to get the list of receptors that contain a certain residue or motif.

For human class C receptors, GPCR-SAS shows that position 3.40 mostly contains an aromatic residue (Tyr:50%, Phe:27.3%; entropy 0.4), but position 6.44 exhibits more variability (Thr:36.4%, Ser:18.2%, Tyr:13.6%, Glu:9.1%, Val:9.1%; entropy of 0.6). The most common pairs of residues at these positions in the class C receptors are aromatic-polar (Tyr-Thr:36.4%, Phe-Ser:18.2%, Tyr-Glu:9.1%) and aliphatic-aromatic (Ile-Tyr: 9.1%). The subfamily of metabotropic glutamate receptors (mGluRs) all contain Tyr3.40 and Thr6.44 (see mGluR5 in Fig 2F). Like class B receptors, class C receptors lack Pro5.50. We next analyzed the covariance of positions 3.40 and 6.44, that is, if changes in position 3.40 occur together with changes in position 6.44. Both GPCR-SAS revealed statistically significant covariance using OMES and MIp analyses (p-values <0.001 in both cases) with Z-scores of 4.28 and 4.78 respectively (Fig 4A). Furthermore, A GPCRs-SAS correlation analysis for aromatic residues at position 3.40 and polar residues at position 6.44 provided an odds ratio of 18.67 (with a 95% confidence interval of 1.50 to 232.3). For better statistics, we increased the number of sequences by using the “All organism” set. In this case we got a stronger association (odds ratio of 40.89) and a narrower confidence interval (13.48 to 123.99), (Fig 4B). Similarly, (with the “All organism set”) we obtain an odds ratio of 28.27 for an apolar residue at position 3.40 and an aromatic residue at position 6.44 (with a 95% confidence interval between 3.67 and 217.83), supporting a coordinated role for both amino acids. This covariance of positions 3.40 and 6.44, together with correlation of specific amino acids at these positions clearly suggest that both residues have a functional role in class C receptors as part of the same molecular switch. In fact, this is in accordance with our recent proposal, based on mutagenesis experiments and molecular modelling, that Tyr3.40 and Thr6.44 are part of the “transmission switch” in the GRM2 [37].

## Conclusions

Here we present GPCRS-SAS, a web application that allows to perform frequency, covariance and correlation analyses for sequence positions or motifs in GPCRs. The tool takes advantage of the structural similarity in the TM domain of GPCRs and allows performing comparisons and statistical analyses of sequence positions or motifs within the TM helices and helix 8 for receptors of classes A, B, C and F. As an example of use, we here show its utility in the successful extrapolation of the “transmission switch” of class A GPCRs to classes B and C. GPCRS-SAS tells that nearly half the class A GPCRs have as triads for positions 3.40–5.50–6.44 either Ile-Pro-Phe (32.3%) or Val-Pro-Phe (16.5%). Class B and Class C receptors lack Pro5.50 and contain alternative, but also conserved, pairs at positions 3.40–6.44. The preferred pairs in class B receptors are Phe-Leu (45.8%) and Tyr-Leu (12.5%). This implies a switch between residue types compared to the same positions in class A. The preferred pairs in class C receptors are Tyr-Thr (36.4%) and Phe-Ser (18.2.1%). Overall, as supported by previous mutagenesis data [28, 37], this analysis suggests that different “transmission switches” involving the same positions but different residue types exist in classes A, B and C, and are likely to represent common activation pieces within the whole superfamily. The type of statistical analyses that GPCR-SAS performs can be used to find functionally important residues or groups of residues undergoing correlated evolution, and thus represents a useful instrument to rationalize ligand selectivity, G protein recognition or receptor activation, among others.

## Supporting information

S1 Fig **The sequence alignment of the TM segments and helix 8 of selected GPCRs from classes A, B, C and F.** The alignment is colored using Clustal scheme. Receptor abbreviations are as in UniProt. Greek letters α, β, γ and δ represent the branches described by Fredriksson and collaborators for the class A. Receptors of the class C do not have helix 8 according to the presently available structures.
(TIF)

S2 Fig Snake-plot representation of the human Class C GPCRs. Each residue is represented with circle with a blue outline the one-letter amino acid code and the Ballesteros-and-Weinstein numbering scheme. The intensity of the blue outline represents the conservation of the position and the intensity of the gray-black letter represents the conservation of the specific amino acid relative to the consensus sequence.
(TIF)
