# Supplementary figures and images for: GPCR-SAS: A web application for statistical analyses on G protein-coupled receptors sequences
Source: PLoS One. 2018 Jul 25;13(7):e0199843. doi: 10.1371/journal.pone.0199843 (PMC6059404; doi:10.1371/journal.pone.0199843)

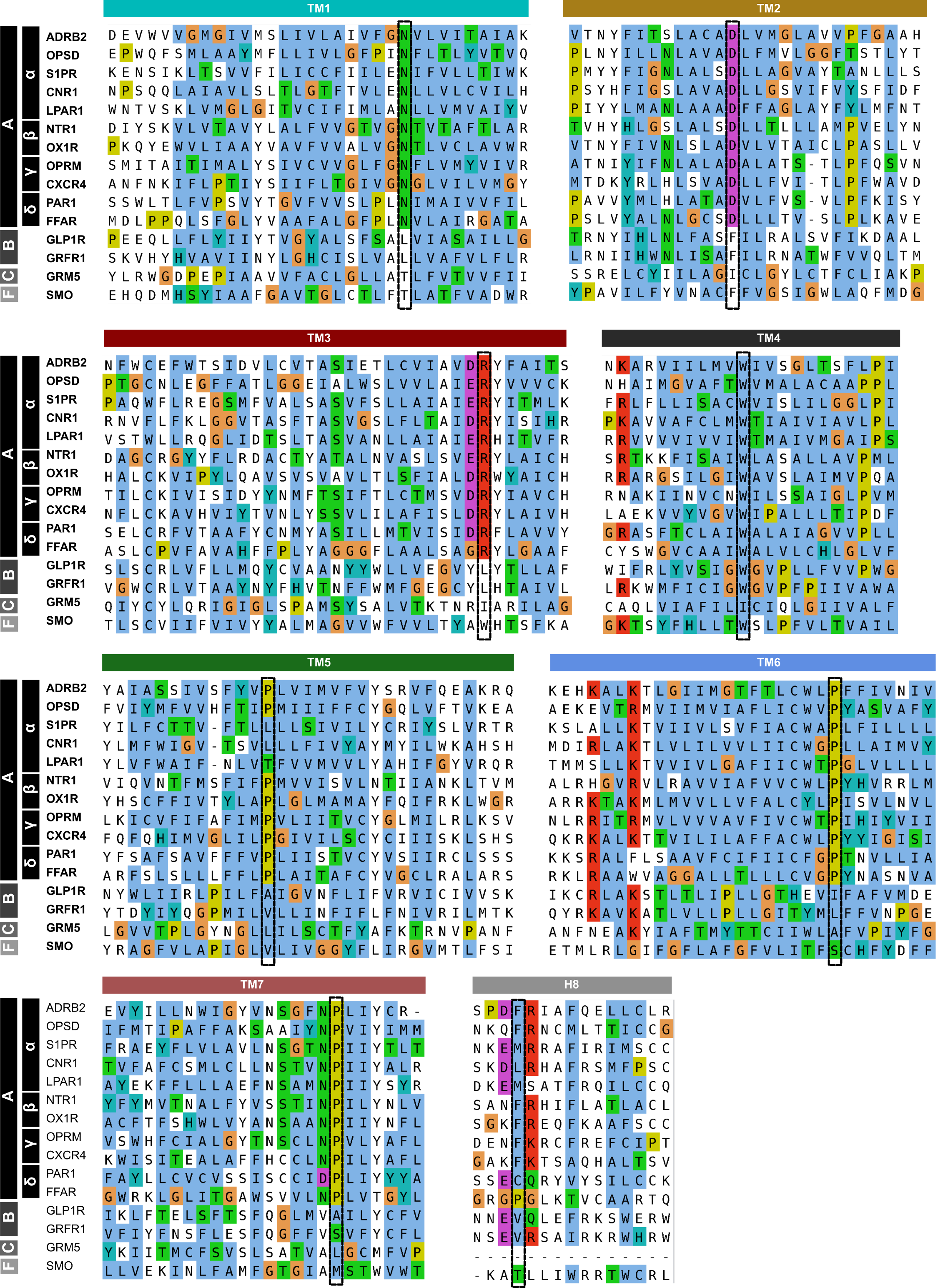

Supplement: S1 Fig — The sequence alignment of the TM segments and helix 8 of selected GPCRs from classes A, B, C and F. The alignment is colored using Clustal scheme. Receptor abbreviations are as in UniProt. Greek letters α, β, γ and δ represent the branches described by Fredriksson and collaborators for the class A. Receptors of the class C do not have helix 8 according to the presently available structures. (TIF) [file pone.0199843.s001.tif]

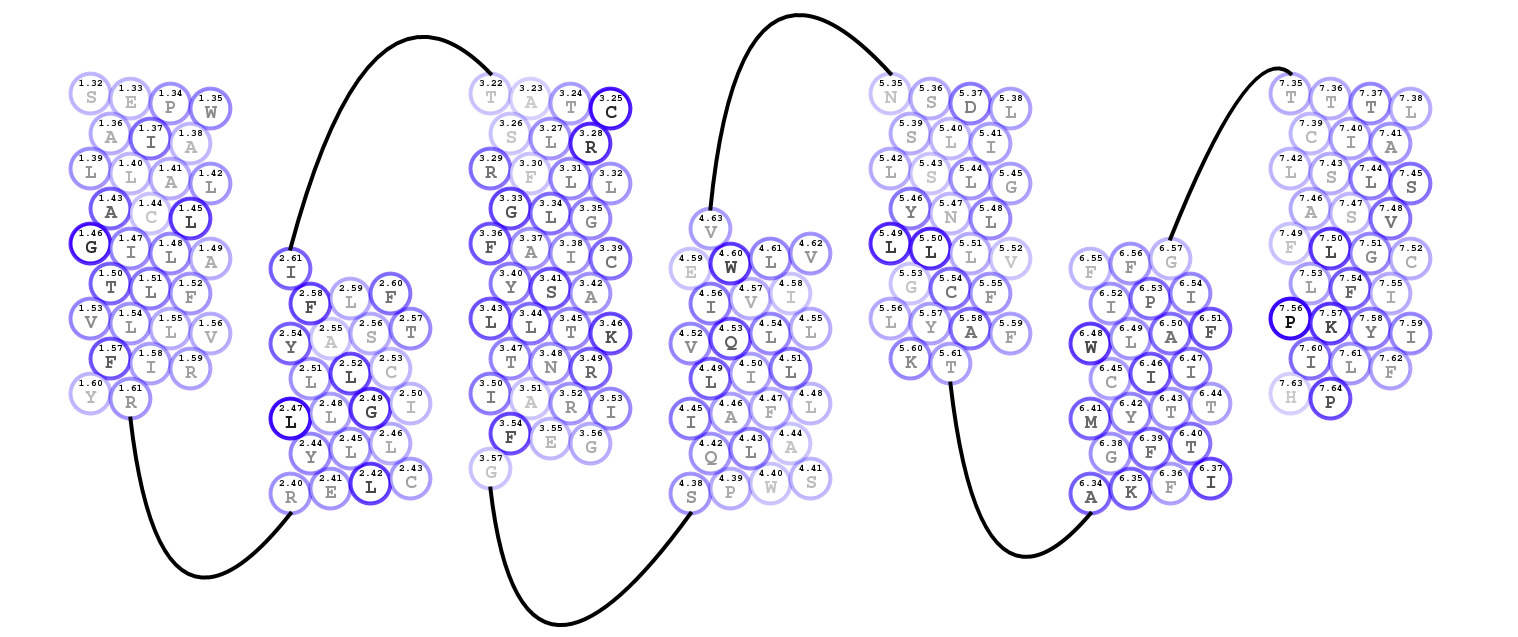

Supplement: S2 Fig — Each residue is represented with circle with a blue outline the one-letter amino acid code and the Ballesteros-and-Weinstein numbering scheme. The intensity of the blue outline represents the conservation of the position and the intensity of the gray-black letter represents the conservation of the specific amino acid relative to the consensus sequence. (TIF) [file pone.0199843.s002.tif]
